# Stabilizing selection on microsatellite allele length at arginine vasopressin 1a receptor and oxytocin receptor loci

**DOI:** 10.1098/rspb.2017.1896

**Published:** 2017-12-13

**Authors:** Phillip C. Watts, Eva R. Kallio, Esa Koskela, Eija Lonn, Tapio Mappes, Mikael Mokkonen

**Affiliations:** 1 Department of Ecology and Genetics, University of Oulu, Oulu 90014, Finland; 2 Department of Biological and Environmental Science, University of Jyväskylä, PO Box 35, Jyväskylä 40014, Finland; 3 Department of Biological Sciences, Simon Fraser University, 8888 University Drive, Burnaby, British Columbia, Canada V5A1S6

**Keywords:** gene dynamics, reproductive behaviour, noncoding genome, VNTR

## Abstract

The loci arginine vasopressin receptor 1a (*avpr1a*) and oxytocin receptor (*oxtr*) have evolutionarily conserved roles in vertebrate social and sexual behaviour. Allelic variation at a microsatellite locus in the 5′ regulatory region of these genes is associated with fitness in the bank vole *Myodes glareolus*. Given the low frequency of long and short alleles at these microsatellite loci in wild bank voles, we used breeding trials to determine whether selection acts against long and short alleles. Female bank voles with intermediate length *avpr1a* alleles had the highest probability of breeding, while male voles whose *avpr1a* alleles were very different in length had reduced probability of breeding. Moreover, there was a significant interaction between male and female *oxtr* genotypes, where potential breeding pairs with dissimilar length alleles had reduced probability of breeding. These data show how genetic variation at microsatellite loci associated with *avpr1a* and *oxtr* is associated with fitness, and highlight complex patterns of selection at these loci. More widely, these data show how stabilizing selection might act on allele length frequency distributions at gene-associated microsatellite loci.

## 1. Introduction

Genes within the vasopressin–oxytocin pathway represent an interesting model of mate choice as they regulate social and reproductive behaviours in diverse taxa [1–3]. Behavioural expression associated with these neuropeptides is often mediated by tissue-specific densities of their receptors, notably arginine vasopressin receptor 1a (V1aR) and oxytocin receptor (OTR) [2,4]. In the prairie vole *Microtus ochrogaster* genome, discovery of an association between the length of a microsatellite within the 5′ regulatory region of the vasopressin 1a receptor gene (*avpr1a*) and brain V1aR density [2,5,6] stimulated research into potential genetic control over mating behaviour. Hence, microsatellite allele length at *avpr1a* is associated with behaviours relevant to mate choice, such as home range size, memory use and partner preference [5,7–9]. Associations between allelic variation at a microsatellite locus in the 5′ regulatory region of *avpr1a* and gene expression, brain receptor density and/or social and sexual behaviour have been found also in chimpanzees and humans [10–15].

Evidence for selection on microsatellite allele length is derived from studies of microtine voles. Prairie vole males with short or long microsatellite alleles at *avpr1a* enjoy greater reproductive success in field experiments [16] and in wild populations [17], respectively, while females with longer *avpr1a* microsatellite alleles produce more offspring [18]. In bank voles, *M. glareolus*, allele length is associated with fitness at the microsatellite locus located in the 5′ regulatory region of *avpr1a*; males with longer *avpr1a* alleles sired more offspring than did males with shorter alleles, but shorter alleles were associated with increased reproductive success in female bank voles [19]. By contrast to *avpr1a*, there is no macroevolutionary conservation of a microsatellite locus in the 5′ regulatory region of oxytocin receptor (*oxtr*). Nonetheless, allelic variation at a microsatellite locus located upstream of the *oxtr* transcription start site in the bank vole is associated with fitness, for example, with shorter *oxtr* alleles increasing male reproductive success [19]. One corollary of these studies is the potential for selection on microsatellite allele length. By contrast, the general processes driving microsatellite allele length distributions emphasize the role of mutation.

**Figure 1.**
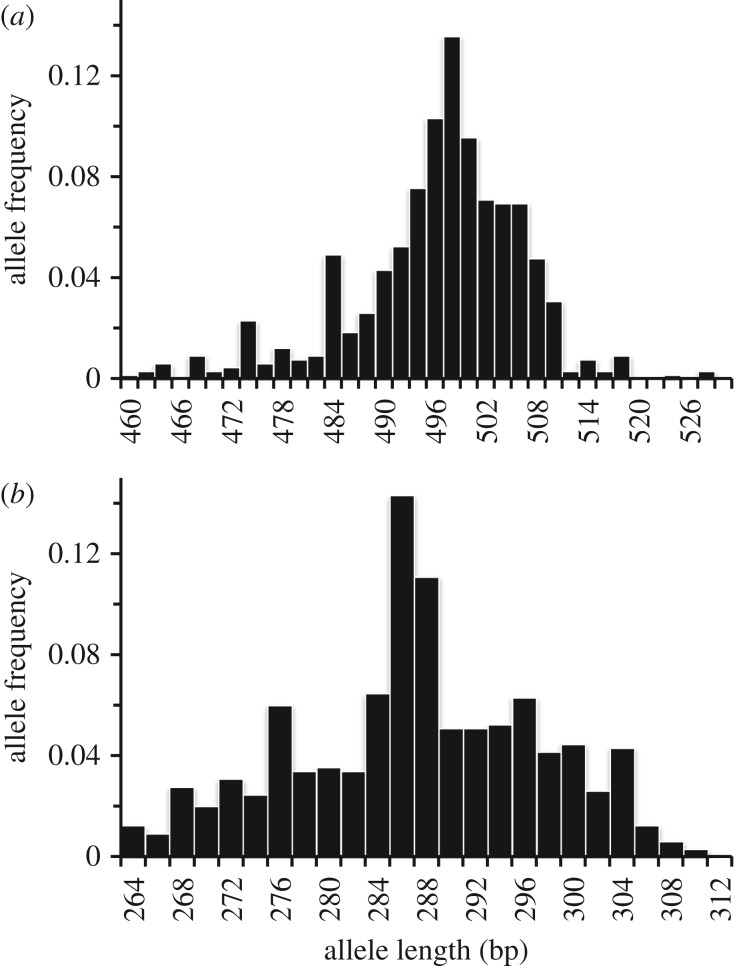
Allele length distribution of microsatellite loci located within the 5′ regulatory regions of (*a*) *avpr1a* and (*b*) *oxtr* in bank voles from central Finland.

Microsatellites have high mutation rates (between 10^−2^ and 10^−6^ mutations per locus per generation) [20,21], with alleles having a tendency to expand in length [21]. Rather than accumulating a random distribution of (longer) alleles, microsatellite loci exhibit (i) distinct upper and lower allele length boundaries and (ii) stationary allele length distributions with low frequencies of the longest and shortest alleles: this characteristic allele length distribution is apparent in wild vole populations at *avpr1a* and *oxtr* (e.g. figure 1; see also [17,22]). Modifications to a stepwise mutation model [23] to constrain allele length include imposing an upper bound to allele length [24,25] or incorporating a size-biased mutation process, where long alleles tend to shorten [26,27] and/or short alleles are biased towards expansion [28,29]. A combination of evidence for fitness effects at *avpr1a* and *oxtr* microsatellite loci and the allele length distribution implies stabilizing selection against the longest and shortest alleles.

To test the hypothesis that selection acts against long and short alleles, we quantified breeding success in bank voles with different microsatellite genotypes at arginine vasopressin receptor 1a (*avpr1a*) and oxytocin receptor (*oxtr*) loci. We concentrate on these loci as they (i) show clear fitness effects in microtine voles and (ii) have evolutionarily conserved roles in vertebrate social and sexual behaviour [1–3]. We find, to the best of our knowledge, the first evidence for selection against both ends of a microsatellite allele length distribution.

## 2. Material and methods

### (a) Microsatellite loci at arginine vasopressin receptor 1a (avpr1a) and oxytocin receptor (oxtr)

The microsatellite within the 5′ regulatory region of *avpr1a* of the bank vole consists of (CA) and (GA) motifs, and is located approximately 920 bp upstream of the *avpr1a* exon 1 [19]; this microsatellite locus is conserved in many rodents, with a microsatellite located at 903, 963, 965 and approximately 980 bp upstream of *avpr1a* exon 1 in the prairie vole (*M. ochrogaster*, Genbank accession number AF069304), mouse (*Mus musculus*, NC_000076), Norway rat (*Rattus norvegicus*, NC_005106) and in eight species of deer mice (*Peromyscus* spp., GU254538-GU254609), respectively. The microsatellite located in the 5′ regulatory region of *oxtr* in the bank vole genome is predominantly (CT) and (GA) motifs located upstream of the putative transcription start site [19]. In *M. musculus*, a microsatellite is located immediately (approx. 10 bp) upstream of *oxtr* transcript variant X1 (XM_006505723) or 1448 bp upstream of the *oxtr* transcription start site (NM_001081147).

### (b) Study animals and breeding trials

The bank vole *M. glareolus* is a small rodent that inhabits forests and fields in the Palearctic, extending its distribution from Europe into western Siberia. Female bank voles defend breeding territories and care for their young, while males do not provision their young; both sexes mate multiply [30].

To (i) quantify natural levels of polymorphisms in the *avpr1a* and *oxtr* microsatellite loci, and (ii) examine the influence of microsatellite allele length on breeding success, we caught 325 wild bank voles from central Finland (62°37′N, 26°20′E). Animals were caught at 20 trapping locations distributed over approximately 100 km^2^, with any two trapping locations up to 4.3 km apart. Each location contained four Ugglan Special multiple-capture live traps (Grahnab, Sweden) located at the corners of a 15 × 15 m^2^ (see [31] for full details of bank vole trapping procedures).

Bank voles were housed in the Experimental Animal Unit, University of Jyväskylä in standard Makrolon Type III cages (43 × 26 × 15 cm) with sawdust and hay for bedding, with food (Labfor 36; Lactamin AB, Sweden) and water ad libitum, at 22°C and on a 16 : 8 h L/D photoperiod (dark period is between 22.00 and 06.00). All animals were implanted with electronic identification microchips (Trovan Unique).

Fitness associated with microsatellite allele length is quantified as ‘breeding success’ (production of some or no offspring) using breeding trials: essentially, failure to produce any offspring after an opportunity to mate is zero fitness. To quantify how microsatellite allele length affects breeding success, we paired a male and a female bank vole with known *avpr1a* and *oxtr* genotypes (see Genotyping) in the same cage for two weeks, after which time the animals were separated and the female was monitored daily for birth. Some of the offspring of successful matings were used in the breeding trials. We kept a pedigree to ensure that potential breeding pairs were not close relatives (siblings, or parents and offspring).

### (c) Genotyping

A small sample of ear tissue (field-caught adults) or the tip of tail (laboratory-reared young) was taken from as a source of DNA. DNA was extracted using the Qiagen DNeasy Tissue kit and a Kingfisher magnetic particle processor (Thermo Fisher Scientific). Primer sequences and thermal cycling conditions to amplify *avpr1a* and *oxtr* microsatellite loci are provided in [19]. PCR products were pooled with a LIZ600 size standard, separated by capillary electrophoresis on an ABI3100 and sized using GeneMapper v. 3.7 (Applied Biosystems).

### (d) Analysis of breeding success

Our aim was to determine whether the microsatellite allele length of an individual affected its breeding success. We examined the probability that animals produced offspring (1 = yes, 0 = no) after an opportunity to mate in relation to microsatellite allele length at *avpr1a* and *oxtr*, as microsatellite allele length had little effect on the litter size of the breeding voles (see electronic supplementary material, for methods and results). Models (described below) were run separately for *avpr1a* and *oxtr* as they are independent loci [19].

Our first analysis considered whether an individual's allele length affected breeding success. We estimated the breeding probability as the proportion of successful matings (i.e. the individual produced offspring) out of *n* breeding trials per individual (proportional outcome) using generalized linear modelling (GLM) with binomial error distribution and logit link function. As many individuals were paired with several partners, the partner's genotype was not considered in this first analysis and the sexes were examined separately. Potential explanatory variables included in the full global model were: (i) MAL—mean (over the diploid genotype) allele length of an individual (centred over the average MAL of all females or all males) and (ii) its polynomial term (i.e. mean allele length^2^), and (iii) DAL—the difference in allele length between the two alleles within an individual, and (iv) the origin of the individual (i.e. wild-caught or laboratory-born). In addition, (v) all two-way interactions were included in the full model. The full model showed some overdispersion (dispersion parameter 1.1–1.7) that was taken into account using quasi-binomial models [32]. Model selection was based on the Akaike information criterion adjusted for sample size using quasi-AICc (QAICc) for model ranking [33]. The most parsimonious model (i.e. the model with the least explanatory variables) within 2 QAICc units from the model with the lowest QAICc was selected as the best model supported by the data [34] and used for statistical inference (see electronic supplementary material, table S1 for model selection). Model selection was carried out using the dredge command in the MuMIn package [35] for R v. 3.3.1 [36].

Our second analysis quantified whether breeding success was associated with the interaction between the genotypes of pairs: whether the combination of microsatellite alleles affected reproduction. For these analyses, we considered only data for the pairs of individuals that had successfully bred a least once in the laboratory. We estimated the breeding probability (binary outcome) using the generalized linear mixed modelling (GLMM) approach implemented by the glmer function in lme4 [37], with a binomial error distribution and logit link function. To control for potential pseudo-replication (from repeated observations per individuals), male and female identities were included as random effects. The full models included the four main effects listed above (i–iv) and (v) the two-way interactions that were identified as significant in the first analysis for both sexes, as well as (vi) the two-way interactions between female and male variables, and (vii) the difference in the mean allele length between the female and male of each pair was included (electronic supplementary material, table S2). As described above, model selection was based on AICc, using the dredge function (in MuMIn) to identify the best model.

## 3. Results

### (a) Microsatellite characteristics

Allele frequency-length distributions at both microsatellite loci show higher frequencies of the intermediate length alleles and lower frequencies of long and short alleles in wild-caught bank voles (figure 1). For *avpr1a*, the long (greater than 512 bp) and short (less than 482 bp) alleles occur at low (less than 0.02) frequencies while the intermediate (496–502 bp) length alleles occur at an allele frequency of approximately 0.10 or higher (figure 1*a*); similarly, at *oxtr* the long (greater than 306 bp) and short (less than 274 bp) alleles occur at lower (approx. 0.02 or less) frequencies than the intermediate length (286–290 bp) alleles (greater than 0.10 allele frequency) (figure 1*b*).

### (b) Associations between microsatellite allele length and breeding success

#### (i) Effect of individuals' own microsatellite length on breeding probability

Data on individual breeding probability represent breeding trials for 952 bank voles (509 females, 443 males), with 1–13 and 1–15 observations per female and male, respectively: in total, the analyses are based on 1310 breeding trials, of which 410 were successful.

**Figure 2.**
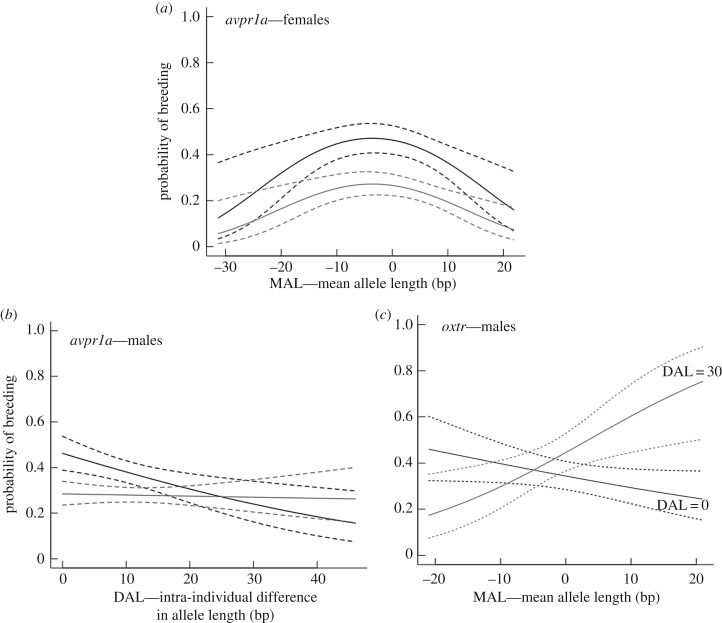
Effect of microsatellite allele length on probability of breeding by bank voles. Probability of individual (*a*) female voles breeding in relation to mean *avpr1a* allele length (MAL) and (*b*) individual male voles breeding in relation to the difference in *avpr1a* allele length within an individual's genotype (DAL). Black lines show data for wild-caught animals and grey lines show data for animals bred in the laboratory. (*c*) Probability of male voles breeding in relation to the difference in mean *oxtr* allele length within an individual's genotype; black line indicates difference in allele length (DAL) = 0, and grey line indicates a DAL of 30 (both cases indicate predicted relationship for wild-caught individuals). Dotted lines show 95% confidence intervals of the means.

An individual's *avpr1a* microsatellite allele genotype is associated with its probability of breeding, with an apparent adverse effect of long and short alleles. Significant effects on female breeding probability were derived from (i) the origin of the individuals (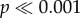), with wild-caught females more likely to breed than those born in the laboratory, and (ii) nonlinear effect of the mean length *avpr1a* alleles (*p* = 0.004) (table 1). A reduced probability of breeding in laboratory animals likely reflects the timing of the breeding trials. Wild-born animals are caught during the start of the breeding season, and while breeding between laboratory-reared animals can continue after the natural breeding season has ended, some animals do not breed. Nonetheless, females whose genotypes comprise *avpr1a* microsatellite alleles towards the average of the length distribution were more likely to breed than were females with long or short microsatellite alleles (figure 2*a*). In males, the best model identified that (i) the individual's origin (*p* < 0.001) and (ii) the interaction between individual's origin and the difference in the length of *avpr1a* alleles in a genotype (*p* = 0.034) had significant associations with breeding probability (table 1); wild-caught, but not laboratory-reared, male bank voles whose alleles were more different in length (i.e. genotypes with a long and a short allele) had lower breeding success (figure 2*b*).

**Table 1.** Final GLMs after model selection that provide the probability of successful breeding (logit scale) by individual bank voles, *M. glareolus*, in relation to the animal's origin (wild-caught or laboratory bred), genotype (MAL—centred mean allele length and its polynomial term or DAL—the intra-individual difference in centred allele lengths) at two microsatellite loci (*avpr1a* and *oxtr*); also shown are significant two-way interaction terms. Successive values for the intercept represent (i) a laboratory-born female with mean allele length (0, corresponds to 495.8 bp), (ii) a laboratory-born male with the difference between the allele lengths = 0 and (iii) a laboratory-born male with the difference between the allele lengths = 0 and centred mean allele length = 0.

locus/gender (sample size) source of variation	estimate (s.e.)	*t*-value	*p*	dispersion parameter
*avpr1a*/female (*n* = 509)				
intercept	−1.015 (0.122)	−8.337	<0.001	
origin (field)	0.869 (0.159)	5.475	<0.001	
MAL	−0.017 (0.010)	−1.670	0.096	
MAL^2^	−0.002 (0.000)	−2.879	0.004	1.656
*avpr1a*/male (*n* = 443)				
intercept	−0.921 (0.132)	−6.994	<0.001	
origin (field)	0.769 (0.202)	3.807	<0.001	
DAL	−0.002 (0.009)	−0.266	0.791	
origin × DAL	−0.031 (0.015)	−2.131	0.034	1.111
*oxtr*/male (*n* = 443)				
intercept	−1.076 (0.116)	−9.264	<0.001	
origin (field)	0.428 (0.131)	3.272	0.001	
DAL	0.014 (0.008)	1.880	0.061	
MAL	−0.023 (0.013)	−1.866	0.063	
DAL × MAL	0.003 (0.001)	2.715	0.007	1.109

No significant associations between *oxtr* microsatellite length and breeding probability were identified when considering a female's genotype in isolation (electronic supplementary material, table S1). In male bank voles, the best model identified significant effects of (i) individual origin (*p* = 0.001) and (ii) the interaction between the difference between *oxtr* allele lengths within an individual and the mean allele length *oxtr* of the individual (*p* = 0.007) (table 1); in effect, male bank voles with longer mean *oxtr* alleles showed a reduced probability of breeding when the difference in length between alleles is small (i.e. two long alleles), while in more heterogeneous (i.e. large difference in allele length) males the probability of breeding increased with increasing mean allele length (figure 2*c*).

#### (ii) Effect of the genotypes of the female–male pairs on breeding probability

In total, there were 546 breeding trials (out of 1310 attempted breeding trials trials) between individuals that had ever reproduced in the laboratory; these pairings involved 220 females and 256 males (with one to eight breeding observations per animal).

Microsatellite allele length showed significant associations with the probability that a pair of animals produced offspring. At *avpr1a*, the probability of breeding by bank voles in relation to their microsatellite length was significantly associated with (i) the origin of the animals (*p* < 0.001 and *p* = 0.020 for females and males, respectively, with wild-caught animals more likely to breed than those raised in the laboratory), (ii) nonlinear mean allele length of the female's genotype (length^2^; *p* = 0.001) (table 2). Thus, only the female's average *avpr1a* microsatellite allele length has an effect on a pair's breeding success, with highest breeding probability occurring around the mean length.

**Table 2.** Final GLMMs after model selection that provide the probability of successful breeding (logit scale) by pairs of bank voles, *M. glareolus* in relation to the origin of the animals (wild-caught or laboratory bred), genotype (MAL—the centred mean allele length MAL and its polynomial term, DAL—the intra-individual difference in allele length, and DAL_FM—the difference in centred mean allele lengths between a potential breeding pair) at two microsatellite loci (*avpr1a* and *oxtr*); also shown are significant two-way interaction terms, the variance attributable to random effect (*σ*^2^) and the standard deviation of *σ*^2^ (s.d.). Intercept for *avpr1a* represents a pair with a laboratory-born male and laboratory-born female, which difference between the allele lengths was = 0 (i.e. no difference in length in the two alleles) and centred mean allele length = 0. Intercept for *oxtr* represents a laboratory-born female with the difference between the allele lengths = 0 (i.e. no difference in length in the two alleles) and difference between the male in the mean *oxtr* length = 0.

				random effect, *σ*^2^ (s.d.)	
locus (sample size) source of variation	estimate (s.e.)	*z*-value	*p*	female	male
*avpr1a* (*n* = 546)					
intercept	0.859 (0.180)	4.767	<0.001		
origin (field: female)	0.733 (0.215)	3.403	<0.001		
origin (field: male)	0.529 (0.228)	2.319	0.020		
MAL (female)	−0.041 (0.014)	−3.007	0.003		
MAL^2^ (female)	−0.004 (0.001)	−3.225	0.001	0.079 (0.28)	<0.001 (0.00)
*oxtr* (*n* = 546)					
intercept	1.712 (0.294)	5.817	<0.001		
origin (field: female)	0.656 (0.218)	3.015	0.003		
DAL (female)	−0.032 (0.013)	−2.539	0.011		
DAL_FM	−0.063 (0.018)	−3.585	<0.001	0.005 (0.07)	0.122 (0.35)

At *oxtr*, a negative effect of divergent alleles was apparent, and the probability of breeding was associated with (i) the origin of the females (*p* = 0.003), (ii) the level of dissimilarity in male and female genotypes (average length of alleles) (*p* < 0.001) and (iii) the level of dissimilarity in the length of a female's own alleles (*p* = 0.011) (table 2). Hence, the association between *oxtr* and breeding probability includes an interaction between male and female genotypes, as well as an effect of the female genotype, with an increase in genetic dissimilarity (either between males and females, or when a female's alleles were different) associated with lower breeding probability (table 2) (see electronic supplementary material, table S2 for model selection).

## 4. Discussion

Expression of arginine vasopressin receptor 1a (*avpr1a*) and oxytocin receptor (*oxtr*) plays important roles in vertebrate social and sexual behaviour [1–3]. By examining an extensive dataset of breeding success of bank voles with known genotypes at microsatellite loci in the regulatory regions of *avpr1a* and *oxtr*, we provide evidence for stabilizing selection against long and short allele lengths. These data show how microsatellite genotypes can impact fitness and allele length distributions at *avpr1a* and *oxtr* microsatellite loci.

### (a) Avpr1a and oxtr microsatellites as fitness loci

Offspring production can be an inherent property of an individual's microsatellite genotype via (i) selection for intermediate length alleles *per se* (females at *avpr1a*) or (ii) selection favouring genotypes that comprise similar length alleles (males at *avpr1a* and females at *oxtr*). How might variation in microsatellite allele length exert tangible fitness consequences? Microsatellite loci located in 5′ regulatory regions can regulate transcription, for example by modifying chromatin structure, overlapping with protein binding sites and/or affecting the spacing of promoter elements [38–40]. Indeed, an association between *avpr1a* microsatellite allele length and gene expression (in specific regions of the brain) has been documented in bank voles [19], prairie voles [2] and in humans [11,13]. While other genomic elements (e.g. single nucleotide polymorphisms, CpG islands) can affect transcription, including expression of *avpr1a* in prairie vole brains [6,41], selection against long and short alleles at both *avpr1a* and *oxtr* indicates a more general role for microsatellite allele length.

A negative effect of both long and short microsatellite alleles broadens the fitness consequences of microsatellite allele length beyond diseases (e.g. Fragile X, Huntington's disease) caused by a major expansion of the repeat array [39,42]. Moreover, we are not aware of any previous report of an individual whose microsatellite genotype comprises dissimilar-length alleles experiencing a reduction in probability of breeding. Mechanisms behind this apparent intralocus conflict are not known, but could reflect ‘poor’ function associated with having a long or short allele (e.g. certain alleles might impair gene expression via an effect on transcription), or a negative interaction between alleles with quite different lengths. Interestingly, the allele length distribution at *avpr1a* and *oxtr* (figure 1) necessitates that animals with particularly long or short alleles have a low probability of finding a potential mate with a similar (or very dissimilar) genotype. Microsatellite allele length is associated also with the probability of breeding between pairs in *oxtr*. Identifying the genetic basis of mate choice is a key issue in evolutionary biology, particularly the relative influence of choice for ‘compatible genes’ [43]. A negative association between breeding success and genetic differences at the *oxtr* microsatellite locus raises a potential for assortative mating at this locus.

### (b) Microsatellite allele length distributions

As outlined previously, hypotheses about the mechanisms that determine microsatellite allele length distributions emphasize mutational processes. One argument against a general effect of selection on microsatellite allele length distributions is a lack of evidence about how subtle differences in length at non-genic microsatellite loci might impact fitness [44]. Here, the definition of non-genic becomes important. Microsatellite loci are seldom translated but nonetheless are enriched within and around coding sequence [45,46] where they can affect transcription [5,38–40] and thus can impact fitness. Our data provide clear evidence that breeding success can be negatively affected by long and short alleles at two microsatellite loci. This potential stabilizing selection on allele length could impact the microsatellite allele length distribution.

Another feature of our data is the negative association between the difference in length of the alleles within an individual and breeding success. Microsatellite alleles paired with dissimilar length homologues are more likely to mutate, which in turn could drive increased heterozygosity [44,47]. Selection against genotypes with particularly different allele lengths might counter this mechanism of increasing mutation rate. Similarly, the mutation rate at microsatellite loci can depend upon allele length [20,48]: selection against long and short alleles raises the potential for an interaction between selection and mutation dynamics.

### (c) Diverse action of selection acting on avpr1a and oxtr microsatellite loci

This study highlights diverse modes of selection at the microsatellite loci associated with the 5′ regulatory regions of *avpr1a* and *oxtr*. For example, the action of directional selection is consistent with studies of prairie voles where either longer [8,17,49] or shorter [16] *avpr1a* alleles were associated with greater reproductive success. The contrast between the results of Solomon *et al*. [16] and Keane *et al*. [17] implies that the direction of selection for *avpr1a* allele length can differ in male prairie voles. Indeed, in the bank vole, the optimum allele length at the *avpr1a* and *oxtr* microsatellite loci depends on an individual's sex and the population density, indicating that balancing selection operates [19]. Under directional or balancing selection, genotypes with either long or short alleles obtain some reproductive benefit and yet our results indicate potential stabilizing selection at the *avpr1a* and *oxtr* microsatellite loci. Several reasons may explain why studies can identify a different mode of selection on *avpr1a* and *oxtr* microsatellite loci. First, some analyses of prairie vole reproductive behaviour partitioned *avpr1a* alleles into short and long categories [9,17,18]; this categorical approach to analysis emphasizes the centre of the microsatellite allele length distribution rather than fitness associated with the longest and shortest alleles. Second, laboratory and field environments can target different components of reproductive success, for example with laboratory studies directed towards mate choice [8] while field experiments often include effects such as intraspecific competition and survival of offspring to weaning [17,19]. Third, any analysis of the reproductive success of wild voles (e.g. [17]) will probably lack sufficient animals (i.e. have low statistical power) to detect selection against the longest or shortest alleles because of the low frequencies of the longest and shortest *avpr1a* and *oxtr* alleles in nature (figure 1) [17,22]. Fourth, some field experiments used animals that were bred in laboratory colonies (e.g. [9,16,19]) and thus reflect patterns after potential stabilizing selection on allele length during laboratory breeding; indeed, the pattern of reproductive success associated with *avpr1a* microsatellite allele length in male prairie voles differed between a field experiment using laboratory-derived animals [16] and an analysis of parentage in a wild population [17]. Finally, our finding that the difference in allele length can affect breeding success would not be apparent from previous analyses of vole reproduction that quantified the fitness of genotypes that differed in mean/summed allele length (e.g. [16,17]) or which measured an intergenerational change in allele frequency [19]. Lonn *et al*. [19] did not release bank voles whose genotypes comprised a long and a short allele into their experimental enclosures, and thus they could not have detected an effect of intra-individual difference in allele length on breeding success. Also, when the difference in allele length among sexes is a component of breeding success, then long or short alleles could be maintained by reproduction with individuals that possess genotypes with intermediate length alleles. In summary, studies of vole reproductive success associated with *avpr1a* or *oxtr* microsatellite genotypes have used different experimental designs and targeted different regions of the microsatellite allele length distribution. At *avpr1a* and *oxtr*, we suggest that stabilizing selection can impact the frequency of alleles at the extremes of a length distribution, while the remaining allelic variation is subject to balancing selection [19]. These complementary field and laboratory studies on rodents show the potential complexity and relevance of selection underlying microsatellite dynamics.

## Supplementary Material

ESM for Watts et al. Stabilising selection on microsatellite allele length

## Data accessibility

Data are available from Dryad: http://dx.doi.org/10.5061/dryad.j7h0c [50].
