# Supplementary material for: Stabilizing selection on microsatellite allele length at arginine vasopressin 1a receptor and oxytocin receptor loci
Source: Proc Biol Sci. 2017 Dec 13;284(1869):20171896. doi: 10.1098/rspb.2017.1896 (PMC5745408; doi:10.1098/rspb.2017.1896)
Supplement: ESM for Watts et al. Stabilising selection on microsatellite allele length [file rspb20171896supp1.pdf]

Electronic Supplementary Material:

Phillip C. Watts, Eva R. Kallio, Esa Koskela, Eija Lonn, Tapio Mappes, Mikael Mokkonen

Stabilising selection on microsatellite allele length at arginine vasopressin 1a receptor and oxytocin receptor loci

Proceedings of the Royal Society of London B, Biological Sciences

DOI: 10.1098/rspb.2017.1896

Supplementary Table S1. Model selection table for probability of breeding (proportional data) predicted by an individual bank vole's *avpr1a* microsatellite allele length characteristics, separately for (a) females and (c) males and for breeding success predicted by an individual's *oxtr* microsatellite allele characteristics, separately for (b) females and (d) males. Variables are: (i) MAL, mean (over the diploid genotype) allele length of an individual (centred over the average mean allele length of all females or males) and (ii) MAL^2, its polynomial term (*i.e.* mean allele length-squared), and (iii) DAL, the difference in allele length between the two alleles within an individual, and (iv) ORIG the origin of the individual (*i.e.* wild-caught or laboratory-born): in addition, (v) all two-way interactions (*) were included in the full model. The full model is underlined, the best model is highlighted bold. All models within 2 QAICc units of the model with the lowest QAICc value are provided. Detailed results of the best models (bold) are provided in the main text and tables therein.

Model	*df*	QAICc	ΔQAICc
a) Female probability of breeding predicted by *avpr1a*			
**ORIG + MAL + MAL^2**	**4**	**740.8**	**-**
ORIG + MAL + MAL^2 + DAL	5	741.1	1.12
ORIG + MAL + MAL^2 + DAL + ORIG*MAL + ORIG*DAL + MAL*DAL	8	747.0	6.28
b) Female probability of breeding predicted by *oxtr*			
**ORIG**	**2**	**729.2**	**-**
ORIG + DAL+ ORIG*DAL	4	729.8	0.57
ORIG + MAL + MAL^2	4	730.6	1.34
ORIG + MAL + MAL^2 + DAL+ ORIG*DAL	6	730.7	1.46
ORIG + DAL	3	730.9	1.67
ORIG + MAL	3	731.1	1.84
ORIG + MAL + MAL^2 + DAL	5	731.2	1.99
ORIG + MAL + MAL^2 + DAL + ORIG*MAL + ORIG*DAL + MAL*DAL	8	734.4	5.27
c) Male probability of breeding predicted by *avpr1a*			
**ORIG + DAL + ORIG*DAL**	**4**	**834.1**	**-**
ORIG + MAL + MAL^2 + DAL + ORIG*DAL	6	834.3	0.14
ORIG + MAL + DAL + ORIG*DAL	5	835.3	1.15
ORIG + MAL + MAL^2 + DAL + ORIG*DAL + MAL*DAL	7	835.5	1.37
ORIG + MAL + MAL^2 + DAL + ORIG*DAL + ORIG*MAL	7	835.7	1.63
ORIG + MAL + MAL^2 + DAL + ORIG*MAL + ORIG*DAL + MAL*DAL	8	837.3	3.16
d) Male probability of breeding predicted by *oxtr*			
ORIG + MAL + MAL^2 + DAL + ORIG*DAL + MAL*DAL	7	835.6	-
ORIG + MAL + MAL^2 + DAL+ MAL*DAL	6	836.0	0.42
ORIG + MAL + DAL+ MAL*DAL+ ORIG*DAL	6	836.3	0.74
ORIG + MAL + MAL^2 + DAL + ORIG*MAL+ ORIG*DAL + MAL*DAL	8	836.8	1.24
ORIG + MAL + MAL^2 + DAL+ ORIG*MAL + MAL*DAL	7	836.9	1.58
**ORIG + MAL + DAL+ MAL*DAL**	**5**	**837.2**	**1.58**

Supplementary Table S2. Model selection table for probability of breeding (binary data) predicted by a bank vole pair's (a) *avpr1a* allele and (b) *oxtr* microsatellite allele characteristics. Variables assessed for model selection include those described in Supplementary Table 1: (i) MAL, mean (over the diploid genotype) allele length of an individual (centred over the average mean allele length of all females or males) and (ii) MAL^2, its polynomial term (*i.e.* mean allele length-squared), and (iii) DAL, the difference in allele length between the two alleles within an individual, (iv) ORIG, the origin of the individual (*i.e.* wild-caught or laboratory-born) and (vi) all significant two-way interactions (*) that were identified in the analysis of individual breeding probability (Supplementary Table 1). Moreover, model selection included (vii) DAL_FM, the difference between the female and male mean allele length as a potential predictor of breeding. Random effects male identity (*n*=256) and female identity (*n*=220) are included in all models. The full model is underlined and the sex of the individual (*i.e.* F_ or M_) is indicated before the variable name. All models within 2 AICc units of the model with the lowest AICc value are provided. The best model is highlighted bold, and the detailed results of the best models (bold) are provided in the main text and tables therein.

Model	*df*	AICc	ΔAICc
a) Pair's probability of breeding predicted by their *avpr1a* characteristics			
**F_ORIG + M_ORIG + F_MAL + F_MAL^2**	**7**	**595.3**	**-**
F_ORIG + M_ORIG + F_MAL + F_MAL^2 + M_MAL + M_MAL^2	9	596.4	1.08
F_ORIG + M_ORIG + F_MAL + F_MAL^2 + F_ORIG*M_ORIG	8	596.4	1.19
F_ORIG + M_ORIG + F_MAL + M_MAL + F_MAL*M_MAL	8	596.6	1.32
F_ORIG + M_ORIG + F_MAL + M_MAL + F_MAL*M_MAL + DAL_FM	9	596.7	1.40
F_ORIG + M_ORIG + F_MAL + F_MAL^2 + M_MAL + F_MAL*M_MAL	9	597.1	1.86
F_ORIG + M_ORIG + F_MAL + F_MAL^2 + M_DAL	8	597.2	1.88
F_ORIG + M_ORIG + F_MAL + F_MAL^2 + M_MAL	8	597.2	1.90
F_ORIG + M_ORIG + F_MAL + F_MAL^2 + F_DAL	8	597.2	1.92
F_ORIG + M_ORIG + F_MAL + F_MAL^2 + DAL_FM	8	597.2	1.94
F_ORIG + F_MAL + F_MAL^2 + F_DAL +M_ORIG + M_MAL + M_MAL^2 + M_DAL + M_ORIG*M_DAL + F_ORIG*M_ORIG + F_DAL*M_DAL + F_DAL*M_MAL + F_MAL*M_DAL + F_MAL*M_MAL + DAL_FM	18	611.1	15.81
b) Pair's probability of breeding predicted by their *oxtr* characteristics			
F_ORIG + M_ORIG + F_DAL + DAL_FM	7	592.8	-
F_ORIG + M_ORIG + F_DAL + DAL_FM + F_ORIG*M_ORIG	8	593.5	0.64
F_ORIG + M_ORIG + F_DAL + DAL_FM + M_DAL	8	594.0	1.16
**F_ORIG + F_DAL + DAL_FM**	**5**	**594.1**	**1.25**
F_ORIG + M_ORIG + F_DAL + M_DAL + DAL_FM + F_ORIG*M_ORIG	9	594.4	1.61
F_ORIG + M_ORIG + F_DAL + DAL_FM + M_MAL	8	594.8	1.93
F_ORIG + F_MAL + F_MAL^2 + F_DAL +M_ORIG + M_MAL + M_MAL^2 + M_DAL + M_MAL*M_DAL + F_ORIG*M_ORIG + F_DAL*M_DAL + F_DAL*M_MAL + F_MAL*M_DAL + F_MAL*M_MAL + DAL_FM	18	608.7	15.90

Supplementary material - analyses of litter size
Methods
We examined whether microsatellite allele length at *avpr1a* and *oxtr* affected an individual's litter size at birth or at weaning (pups at the age of 20 days). Both female and male microsatellite genotypes were examined simultaneously. Separate models were run for *avpr1a* and *oxtr* as they are independent loci. In these analyses we included only successful pairings, *i.e.* 410 litters, of which 402 litters were monitored until weaning.

We estimated the litter size (count variable) using generalized linear mixed model (GLMM) approach with negative binomial error distribution and log link function (glmmadmb command in glmmADMB package in R). The lack of zeros in the litter data (at birth) was taken into account using zero truncated models. To control for potential pseudoreplication (from repeated observations per individuals), male and female identities were included as random effects in the models. The explanatory variables included in the full model were: (i) MAL - mean (over the diploid genotype) allele length of an individual (centred over the average MAL of all females or all males) and (ii) its polynomial term (*i.e.* mean allele length^2), and (iii) DAL - the difference in allele length between the two alleles within an individual, (iv) the difference in the MAL the female and male of each pair, and (v) the origin of the individual (*i.e.* wild-caught or laboratory-born). In addition, (vi) the order of the litter (1st, 2nd, 3rd etc) for the female and for the male and their polynomial terms as well as biologically relevant two-way interactions were included in the full model. The litter size at the age of 20 days (at weaning) was examined using the same explanatory variables, random effects and the model selection procedure as above, but, as some litters had no surviving pups, we used negative binomial distribution without zero truncation. The model selection was carried out manually by omitting one by one non-significant terms, starting from interactions, then polynomials and last non-significant main effects. The model selection procedure was guided by AIC that confirmed that omitting terms decreased the AIC values. The models were simplified until the final models included only significant terms and further simplifying would have increased the AIC value by more than 2 units.

Results
The final model for the litter size at birth, both for *avpr1a* and *oxtr,* included only the origin of the female, with wild captured females having 11 % bigger litters than lab-born females at the time of birth (Table S3). There was no evidence that litter size at birth was affected by microsatellite allele length or other microsatellite characteristics.

Similarly, litter size at the age of 20 days was not associated with *avpr1* microsatellite characteristics but the origin of the female, with wild capture females having 18.2% bigger litters at weaning age than lab-born females (Table S3). The within individual difference in the female's *oxtr* microsatellite length had a significant negative effect on litter size with one base pair increase in the difference associated with a decrease in litter size by 0.8% and the field captured females having 17% bigger litters at weaning age than lab-born females (Table S3).

Supplementary Table S3. Final GLMMs (after model selection) that provide the litter size (log scale) of pairs of bank voles in relation to the origin of the animals (wild-caught or laboratory bred), DAL – the intra-individual difference in allele length, at two microsatellite loci (*avpr1a* and *oxtr*. The variance attributable to random effect (σ^2) and the standard deviation of σ^2 (SD). Intercept for *avpr1a* represents a laboratory born female. Intercept for *oxtr* represents a laboratory born female with the difference between the allele lengths=0 (*i.e.* no difference in length in the two alleles).

Locus (sample size)				Random effect, σ^2 (SD)	
Source of variation	Estimate (SE)	z-value	*P*	Female	Male
Litter size at birth					
avpr1a and *oxtr* (*n*=410)					
intercept	1.253 (0.041)	30.40	<0.001		
origin (field: female)	0.105 (0.053)	1.97	0.049	5.491e-07 (0.0007)	1.248e-07 (0.0004)
Litter size at weaning (20 days)					
avpr1a (*n*=402)					
Intercept	1.166 (0.043)	27.21	<0.001		
origin (field: female)	0.167 (0.055)	3.06	0.002	0.002 (0.0003)	1.128e-07 (0.0004)
oxtr (n=402)					
Intercept	1.265 (0.058)	21.72	<0.001		
origin (field: female)	0.157 (0.054)	2.88	0.004		
DAL (female)	-0.008 (0.003)	-2.40	0.016	5.442e-07 (0.0007)	1.331e-07 (0.0004)
